# Pouting

**Published:** 1859-12

**Authors:** 


### POUTING.

A little girl after playing awhile with some other children, suddenly broke up the happiness of the company by going home in a passion, because "they wouldn't play my way." This may be set down to mere waywardness in a child, but there is much of it in grown persons, in the great plays of practical life. There are persons of education, and culture, and of real benevolence, who refuse their co-operation in a good direction, simply because things are not done precisely in the way to suit themselves, and they go off to whine, and pout, and complain, pouring out there jeremiades in voluble profusion, or to waste their energies in crude experiments, or in impossible endeavors.

The "twelve," of olden time, while yet children in the knowledge of the kingdom, complained to the Master of another because "he followeth not us," is not of our "set," does not belong to our party, is not a member of our church, we are not acquainted with him, we know nothing about him. "Forbid him not" was the broad, and brotherly, and suggestive reply, embodying a generous principle of action for all time. Whoever does good, let him do it, bid him "God speed," "forbid him not."

Some men lack ballast, self-willed, self-confident, and with much apparent benevolence, are at the bottom ugly hearted. If a house is burning down, they will let it burn unless the fire is extinguished in precisely the way which suits them. There is a case in point where some New York gentlemen encouraged the establishment of a "coffee house," where any one could step in and take a cup of coffee at a small cost, with the privilege of reading useful books and newspapers at the same time, without additional expense, the object being to provide a substitute for drinking saloons, bar rooms and beer houses. It would scarcely be supposed that there could be any objection to such a plan. Yet it is opposed, and a substitute is offered thus: "Let us have reading rooms by all means, with pure cold water to give away, but nothing whatever to sell, and paintings and music free to all well behaved persons of both sexes; the young ladies would attract the young gentlemen, and perhaps *vice versa*, without the aid of coffee." They oppose a literal coffee house, because they do not consider it radical enough; it is not the way they would go about exterminating intemperance, for possibly, in their cold water fanaticism, they would contend that drinking coffee is almost as bad as drinking brandy.

Whenever we see a man doing good, let us regard him with a liberal spirit; let us take it for granted that his motives are good, and "forbid him not," simply because he is not of the same set, or sect, or party with ourselves. Let us help him on in so far as he is doing good, and still condemn the evil, for no man's conduct is wholly free from errors in principle, and practice too. So that if the system is rigidly enforced, that a man must be forbidden doing good, because his company, his motives, his principles do not precisely accord with our own, then must all good doing cease, and darkness, and moral death will settle over a ruined world.

There is no more striking exhibition of human presumption and illiberality, than to malign any man, or body of men, to pervert their whole conduct, and make the good they do evil spoken of, simply because in some feature of their operations, there may be in the pouter's opinion, a lack of wisdom or consistency.

"We forbade him because he followeth not us." Here is human nature in one of its narrowest phases; a feature which in the world's history has hindered much of good doing, thwarted much, antagonised much, the doers wherein will have much to answer for in the great day of reckoning. Let all then work with good workers as far as they can, and not speak evil of their good. Where we cannot enforce total abstinence, let us do what we may to encourage a wise moderation.

